# Deadly Threads in the Sky: A Forensic and Public Health Analysis of Kite String-Related Injuries and Fatalities in Northern India

**DOI:** 10.7759/cureus.91233

**Published:** 2025-08-29

**Authors:** Jaspinder Pratap Singh, Sunny Basra, Preeti Chowdhary, Palak Sharma, Arashpreet Kaur, Shubham, Sant Kaur

**Affiliations:** 1 Forensic Medicine, Shri Mata Vaishno Devi Institute of Medical Excellence, Katra, IND; 2 Forensic Medicine, Guru Gobind Singh Medical College, Faridkot, IND; 3 Obstetrics and Gynaecology, Kalpana Chawla Government Medical College and Hospital, Karnal, IND; 4 General Physician, Department of Health and Family Welfare, Amritsar, IND; 5 General Practice, Department of Health and Family Welfare, Ayushman Arogya Kendra Focal Point Jalandhar, Jalandhar, IND; 6 Neuropsychiatry, Bhatia Neuropsychiatric Hospital, Amritsar, IND; 7 Medicine, Government Medical College, Amritsar, Amritsar, IND

**Keywords:** festival-related health hazards, kite string injuries, manjha deaths, manjha-related trauma, public health

## Abstract

Introduction: Kite flying is a popular recreational activity in many parts of the world, particularly in South Asia, including India. The sharp strings can lead to severe neck, face, and upper limb wounds, while in extreme cases, they may result in fatal vascular damage or airway compromise. This study analyzes the pattern, severity, and outcomes of kite string-related injuries over two years at a tertiary center, highlighting the urgent need for regulatory enforcement and public health interventions.

Materials and methods: This retrospective, observational study was conducted at the department of forensic medicine and toxicology in collaboration with the emergency department of a tertiary care center in Northern India. The study covered two years, from January 2022 to January 2024. All cases of kite string-related injuries presenting to the emergency department or referred for medico-legal examination during this time were included.

Results: Most patients were males, accounting for 88.5% (23 out of 26), while only 11.5% were females. The anatomical distribution of injuries highlights the varied severity and patterns across different body regions. The post-mortem findings in the fatal cases reveal the severe and often lethal consequences of kite string injuries.

Conclusion: Kite flying, while being culturally treasured, is a life and limb-preventable risk, particularly with the utilization of unsafe materials. This study reinforces the significance of using forensic results in public health policy to reduce festival-related mortality and injury, thereby making celebrations safe and festive.

## Introduction

Kite flying is a recreationally desired and culturally important activity throughout South Asia, but especially so in Northern India, where the sport is a favorite pastime during Makar Sankranti and Basant Panchami festivals [1]. The use of sharp, abrasive, and chemically treated strings, popularly referred to as manjha, has, however, transformed this otherwise harmless recreation into a serious public safety concern. The strings, traditionally coated with metallic particles or glass powder, have the potential to inflict serious injuries ranging from trivial cuts to deep wounds, vascular sequelae, and even fatality [2]. Motorcyclists, cyclists, pedestrians, and innocent children form the most susceptible group, prone to sustaining injuries in the neck, facial region, and extremities [3-5]. Despite the increased reported cases in urban and rural communities, available literature is largely comprised of isolated case reports or small case series, leading to an enormous paucity of detailed epidemiological information [6-8]. The epidemiology of kite-string-related injuries demonstrates a growing burden, with reports from India, Pakistan, and Bangladesh documenting dozens of deaths and hundreds of injuries annually during kite-flying festivals [4,8,9]. Epidemiological evidence from western India indicates that most victims are two-wheeler riders or pedestrians, with the neck being the most vulnerable site of injury. Many of these incidents occur in January, coinciding with festive kite-flying events [10]. On peak festival days, emergency services in western India have documented an extraordinary surge in patient load, with thousands of mishaps and multiple deaths linked to kite flying within a single day [11]. Reports from central India further highlight injuries caused by Chinese manjha, where even apparently trivial cuts to the hands concealed deeper structural damage requiring surgical care [12]. Case series from northern regions describe some of the most severe outcomes, including traumatic brain injuries, cervical vascular injuries, and airway compromise, with patients occasionally requiring tracheostomy or succumbing to their wounds [13]. The present study aims to analyze two years of injury data from a tertiary care center to delineate demographic patterns, clinical characteristics, and medico-legal implications. It further seeks to identify contributory risk factors and mechanisms, thereby providing evidence to guide preventive legislation, regulatory measures, and public health awareness initiatives.

## Materials and methods

This two-year observational retrospective study was conducted to analyze the epidemiological, clinical, and forensic aspects of kite string injuries. The study was conducted in a Northern Indian tertiary care teaching hospital from January 2022 to January 2024. Ethical clearance was obtained from the Institutional Ethics Committee, Government Medical College, Amritsar, and the study followed well-established ethical norms.

Research methodology

The study was a collaborative effort between the departments of emergency medicine and forensic medicine. It sought to record and analyze the fatal and non-fatal cases of injury brought about directly by kite string contact, focusing on patterns of anatomical injury, causative mechanisms, and outcomes.

Sampling

A systematic listing approach was used. The records of all patients who presented to the emergency department or underwent a medico-legal autopsy due to suspected string injuries of a kite string type during the given research period were screened for eligibility. All the cases of kite string injuries reported in the hospital that were taken into consideration were already earmarked in the record. Data were retrieved through medical records, emergency registers, and autopsy records.

Inclusion and exclusion criteria

Inclusion Criteria

Patients of any gender or age who sustained injuries due to kite strings were included in the study. The mechanism of injury was specifically considered when it involved direct entrapment or collision with a kite string, including synthetic, metallic, or chemically treated strings such as manjha. Cases of fatalities that underwent medico-legal post-mortem examinations, where kite string injury was identified as either a contributory or the sole cause of death, were also taken into account. Furthermore, only those cases with complete clinical records or post-mortem files were included for analysis.

Exclusion Criteria

Accidents that involved injuries resulting from falls, collisions, or kite-flying incidents but not directly attributable to kite strings were excluded from the study. Similarly, cases with an indeterminate etiology or a questionable history of trauma were not considered. In addition, victims with missing or unobtainable medico-legal or clinical records were excluded from the analysis.

Data extraction

Retrospective data were collected from emergency department registers, medico-legal case files, inpatient case records, and autopsy reports (for deceased persons). A total of 26 cases were selected for the study after using the inclusion and exclusion criteria, out of which two were fatal cases, and an autopsy was conducted in those cases. The following variables were defined: demographic factors (gender, age, occupation), type of injury (laceration, incised wound, amputation, vascular compromise), location of the injury, type of kite string used (if applicable), treatment plan, hospitalization status, and clinical or forensic outcome.

Data analysis

The collected data were analyzed through Microsoft Excel 2021 (Microsoft Corporation, Redmond, WA) and then further analyzed through IBM SPSS Statistics for Windows, version 28.0 (IBM Corp., Armonk, NY) [14]. Descriptive statistics were used to report important findings. Percentages and frequencies were obtained for categorical variables, and results were reported in tabular form where relevant. Inferential analysis was not carried out due to the study's small sample size and observational nature.

## Results

Table 1 shows that most of the patients were males, accounting for 88.5% (23 out of 26), while only 11.5% were females (three out of 26). The most affected age group was 31-40 years, contributing 46.2% of the cases, followed by the 21-30 years group (23.15%). No females were affected in the <20, 31-40, 51-60, or >60 years categories.

**Table 1:** Age-wise and gender-wise distribution of the patients.

Age group (in years)	Males	Females	Total (%)
	Number (%)	Number (%)	
<20	01 (3.85%)	00	01 (3.85%)
21 – 30	05 (19.3%)	01 (3.85%)	06 (23.15%)
31 – 40	12 (46.2%)	00	12 (46.2%)
41 - 50	02 (7.65%)	02 (7.65%)	04 (15.3%)
51 – 60	01 (3.85%)	00	01 (3.85%)
>60	02 (7.65%)	00	02 (7.65%)
Total	23 (88.5%)	03 (11.5%)	26 (100%)

Table 2 depicts the anatomical distribution of injuries and highlights the varied severity and patterns across different body regions. The head and face were commonly affected areas, with incised wounds (IW), abrasions (AB), and lacerated wounds (LW) being observed. These injuries primarily involved superficial structures such as the skin and subcutaneous tissue. However, the neck region demonstrated a more severe injury pattern, with damage extending to deeper structures, including muscles and blood vessels, making this site particularly vulnerable to life-threatening outcomes. The upper and lower limbs also showed multiple incised wounds, with deeper tissue involvement, including muscles, tendons, and vessels, suggesting the potential for significant blood loss and functional impairment.

**Table 2:** Pattern of injuries concerning their type, location, and structures damaged. IW: incised wound; LW: lacerated wound; AB: abrasion; s/c: subcutaneous.

S. No.	Site of injury	Type of injury	Underlying structures damaged
1.	Head	IW, AB, LW	Skin, s/c tissue
2.	Face	IW, AB	Skin, s/c tissue
3.	Neck	IW, AB, LW	Muscles, vessels
4.	Upper limbs	IW	Muscles, s/c tissue
5.	Thorax and abdomen	AB	Skin
6.	Lower limbs	IW, AB	Muscles, tendons, vessels

**Figure 1:**
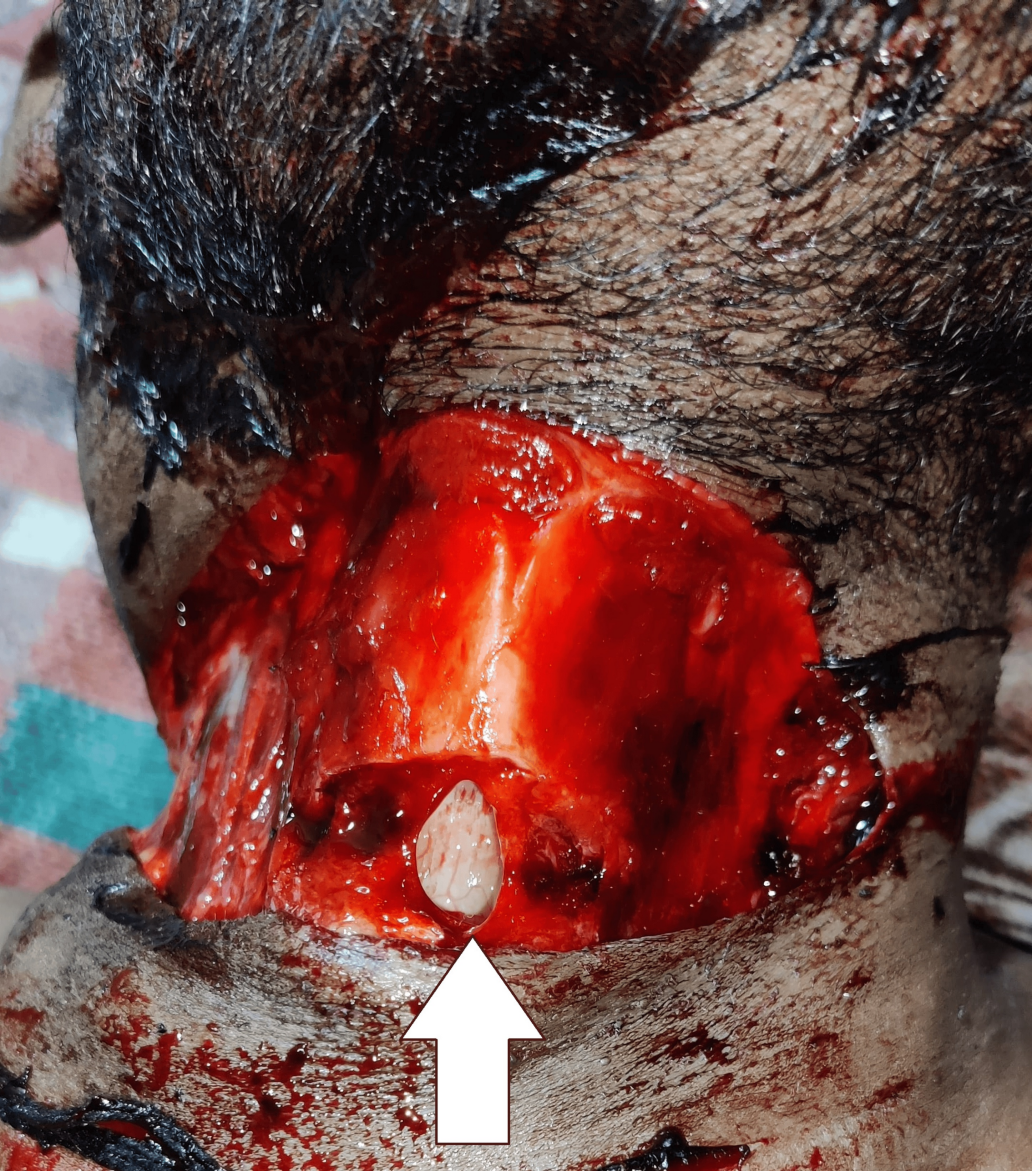
Fatal cut-throat injury with a kite string.

Table 3 represents the post-mortem findings of the fatal cases, revealing the severe and often lethal consequences of kite string injuries. Both victims were young males, aged 27 and 28 years, who succumbed to their injuries within minutes, underscoring the rapid and catastrophic nature of such incidents. The documented causes of death were hemorrhagic shock and cut throat injury (Figure 1), both resulting from extensive blood vessel damage in the neck region. The time since death (TSD) in both cases was recorded as 12-24 hours, indicating that the autopsies were performed within a day, ensuring accurate post-mortem assessments. The findings highlight the fatal potential of kite string injuries, particularly when major vascular structures are compromised.

**Table 3:** Post-mortem findings in fatal cases. M: male; TSD: time since death.

S. No.	Age	Gender	Time between injury and death	Cause of death	TSD
1	28	M	Within a few minutes	Hemorrhagic shock	12-24 hours
2	27	M	Within a few minutes	Cut-throat injury	12-24 hours

## Discussion

Manja string-related injuries, characterized by wounds from manja (i.e., kite string coated with abrasive material such as ground glass, metallic particles, or adhesive), pose a unique scenario of public health significance and medico-legal interest. They can look spontaneous and happen within particular seasons, yet they do possess principal medico-legal value due to their potential for causing serious harm or death.

As per Table 1, which depicts the highest male predominance (88.5%), we have found that the results of our study are consistent with the same pattern found in earlier studies. Nakade et al. (2020) [15], Jarwani et al. (2020) [16], and Patil et al. (2024) [12] have also found that the gender disparity is due to more outdoor activity, kite flying, and motorcycle riding by males, especially during the festival season. Our study's most affected age group (31-40 years) could be the most active age group participating in kite flying or being exposed to traffic injury as commuters. This is in contrast to some studies that consider younger age groups to be vulnerable, like Vaishnav et al. (2019) [11] and Patil et al. (2024) [12]. The results of the present study suggest that the working-age group may be indirectly affected due to occupational exposure, unawareness, or distractions during commutes. The absence of victims in the female group in some age groups may indicate lower participation in kite-related activities or more indoor lifestyles, thus uncovering patterns of gendered exposure. The pattern of trauma is noteworthy in a forensic context.

The anatomical distribution of injuries highlights the varied severity and patterns across different body regions. (Table 2). The upper and lower limbs also showed multiple incised wounds, with deeper tissue involvement, including muscles, tendons, and vessels, suggesting the potential for significant blood loss and functional impairment. The thorax and abdomen, in contrast, sustained more superficial injuries, primarily in the form of abrasions affecting only the skin. Similar findings were observed in the study conducted by Prajapati et al. (2017) [10] and Muvalia et al. (2019) [17]. These superficial but disfiguring injuries indicate the mechanism of horizontal tension by stretched manja, akin to high-velocity interactions, such as collisions with inapparent strings during a two-wheeler commute or inadvertently walking into suspended strings.

On the other hand, the neck area displayed disproportionately severe injury patterns. Profoundly incised wounds that involve both vascular and muscular elements, as seen in the fatal cases in question, have profound clinical and forensic implications. Identification of cut-throat wounds with vascular transection suggests the use of high-tension strings and significant force, usually unexpected by the victim. This finding agrees with the findings of Ganvit et al. (2024) [18], Bhatia et al. (2020) [19], and Vaishnav et al. (2019) [11], who reported life-threatening damage to both vascular and laryngotracheal structures. These injury patterns highlight the neck's vulnerability due to anatomical exposure, the presence of vital structures, and the lack of bony protection.

The post-mortem findings in the fatal cases reveal the severe and often lethal consequences of kite string injuries (Table 3). The findings highlight the fatal potential of kite string injuries, particularly when major vascular structures are compromised. Manja is a highly tensile medium that can cause lacerations with slight contact due to its abrasive covering. While moving (walking, running, bicycling), kinetic energy develops and contributes to the severity and depth of the injuries that are caused. In some cases, this causes a "guillotine effect" on soft tissue, particularly of the neck or extremities. The "guillotine effect" of kite strings aptly describes the sharp, linear incised wounds that occur when abrasive threads, commonly reinforced with glass powder or metallic particles, strike exposed body parts at considerable speed. This mechanism has been well documented in forensic and clinical literature, where victims have presented with deep neck lacerations, transection of major vessels, and, in some instances, fatal airway compromise [3,4,6-8]. Recognition of this effect not only adds to the understanding of injury patterns associated with kite flying but also emphasizes its medico-legal significance and the urgent need for preventive public health measures.

Our experience of more severe limb injuries involving tendons and vessels also aligns with the mechanism of rotational entanglement or defensive movements. Bhatia et al. (2020) [19] reported such injuries where innocent pedestrians had tendon ruptures, contributing to the functional morbidity of such injuries.

Although they are preventable and predictable, string injuries are commonly overlooked. Seasonal peaks have been well reported during festivals like Makar Sankranti and Basant Panchami; however, implementation and enforcement of preventive legislation significantly differ from region to region. Vaishnav et al. (2019) [11] emphasized that protective gear usage is uncommon. Hence, both the participants in kite flying and passersby in the vicinity are at risk of incurring serious or even fatal injuries.

Medico-legally, these are challenging cases. Evidence of trauma, photographs, and correlation with the location or scene (e.g., presence of manja and witness statement) are required to determine the cause and manner of death. Forensic examination must also exclude criminal negligence, particularly in motorcyclists without helmet protection or children flying kites alone. The forensic pathologist must be ready to give an expert opinion on such cases.

In contrast to the results of Nakade et al. (2020) [15], with no mortality, and Vaishnav et al. (2019) [11], with transient airway complications with no deaths, our study reveals a high level of damage severity leading to fatalities. In addition, Jarwani et al. (2020) [16] and Ganvit et al. (2024) [18] had mortality in a few percent of their patients, revealing that although rare, fatalities do occur and should be given much consideration.

Furthermore, the reported evidence by Swain (2022) [20] for the initial treatment in emergency departments, including airway protection, hemorrhage control, and surgery, emphasizes the necessity for expedited triage procedures in suspected kite string injuries. Moreover, the finding of suprastomal granulations or vocal cord paralysis in previous studies points to long-term complications that must be cautiously considered.

Public health implications and preventive strategies in the Indian context

The rising trend of injuries from kite string, particularly those inflicted by lethal "manjha," is a new public health issue in India that needs urgent and multi-faceted intervention. While the cultural and entertainment value of flying kites cannot be disputed, the transition from the traditional cotton strings to artificial, glass-coated, or metal-strengthened ones has transformed the hobby into a dangerous activity with significant morbidity and mortality. The burden is woefully high in the form of celebrations like Makar Sankranti, Basant Panchmi, Uttarayan, and Independence Day, reflecting this public health hazard's seasonal and region-specific pattern [21,22].

One of the most vulnerable groups affected by kite string injuries in India is two-wheeler riders, who are at high risk due to the exposed nature of their mode of transport. Kite strings hanging across roads can entangle riders' necks or faces at high speeds, leading to deep incised wounds, vascular lacerations, and, in severe cases, decapitation or instant death [23]. To mitigate such risks, it is imperative to introduce and popularize specially designed protective gear for two-wheeler users. This could include modified helmets with integrated neck guards, transparent face visors extending below the chin, and frontal mesh attachments or guards, which are usually inverted U or V-shaped metallic guards that can be installed at minimal cost on vehicles to deflect entangling strings. These engineering solutions could function as effective passive preventive measures [24].

Public policy and legislative enforcement must play a central role in harm reduction. Notably, the Chandigarh Government officially banned glass-coated manjha in 2022 following an appeal by the People for the Ethical Treatment of Animals (PETA) India, citing risks to both human and animal life [25]. However, enforcement remains inconsistent across states, with illegal sales and usage continuing unabated, primarily through unregulated local vendors and e-commerce platforms. Stronger interdepartmental coordination between urban municipal bodies, police, pollution control boards, and wildlife departments is crucial to implementing these directives uniformly and effectively [26].

Community-based campaigns also play a significant role. Public service announcements, school-based educational posters, and pre-festival sensitization campaigns can be employed to raise awareness regarding the harm caused by synthetic manjha usage. Messages need to be directed toward kite flyers, shop owners, and parents to discourage harmful materials and encourage safer options such as biodegradable cotton threads. Social media and civil society organizations can be utilized to maximize the outreach and effectiveness of such campaigns [11].

Public health strategies must also encompass awareness campaigns through mass media, schools, and community programs. These campaigns should educate the public, especially children, teenagers, and parents, about the dangers of using unsafe kite strings and the importance of responsible kite flying. School-based interventions during the festival season could effectively instill safety practices among young kite enthusiasts. In addition, real-time surveillance systems in emergency departments and municipal hotlines can be deployed during festival periods to monitor and respond swiftly to kite-related injuries. Notably, several city police departments across India, such as in Delhi, Hyderabad, and Ahmedabad, have employed drone-based aerial surveillance during kite-flying festivals. These drones help law enforcement monitor rooftops, public gatherings, and areas prone to illegal kite string use, enhancing regulatory enforcement and enabling swift intervention. Integrating such technology-driven surveillance with public health and civic administration can significantly reduce injury incidence and facilitate better implementation of bans and safety guidelines [27].

Furthermore, the environmental and ecological dimensions of synthetic manjha injuries cannot be ignored. Birds, particularly raptors and pigeons, often fall prey to entanglement, resulting in mutilation or death. This ecological harm underscores the broader systemic risks of such recreational practices, warranting a multi-sectoral response. Similarly, in a significant judicial development, the Telangana High Court, in 2024, reinforced the 2017 National Green Tribunal (NGT) directive mandating all Indian states to ban the sale, storage, and use of synthetic manjha. At the policy level, integrating kite-string injury data into national injury surveillance systems will allow more effective targeting of healthcare resources and the generation of targeted injury prevention guidelines. Health department, city government, and law enforcement agency coordination can facilitate an effective multi-sectoral response strategy [28,29].

Finally, there needs to be capacity building of the health workers to manage such cases of trauma effectively. Emergency departments in high-incidence areas must be trained in diagnosing and managing kite-string injuries, including immediate airway management, hemorrhage control, and medico-legal documentation protocols. This study presents autopsy-based evidence as a critical feedback mechanism for public health managers to readjust interventions against mortality patterns.

## Conclusions

This retrospective analysis offers a key forensic and clinical understanding of the frequency, severity, and lethal potential of manjha-induced injuries due to kite string in Northern India. The higher proportion of adult males within the working age group among victims highlights the occupational and gender-related vulnerability to the injury. Most events occurred in the context of celebrations during seasonal festivals, thereby highlighting the temporal co-association of cultural rituals with avoidable trauma. Of particular concern, the fatal cases, each with catastrophic neck trauma, highlight the deadliness of glass-coated and chemically treated manjha, necessitating urgent regulatory action and public health response.

However, observation is confounded by single-center study settings, limited numbers of patients involved, and retrospection, possibly compromising its generalizability. Additionally, the lack of elaboration on behavior, environment, and socioeconomic details limits detailed epidemiological analysis of risk factors and prevention measures. Kite flying, while being culturally treasured, is a life and limb-preventable risk, particularly with the utilization of unsafe materials. This study reinforces the significance of using forensic results in public health policy to reduce festival-related mortality and injury, thereby making celebrations safe and festive.
